# Unusual Cushing's Syndrome and Hypercalcitoninaemia due to a Small Cell Prostate Carcinoma

**DOI:** 10.1155/2016/6308058

**Published:** 2016-12-01

**Authors:** Antonio Balestrieri, Elena Magnani, Fiorella Nuzzo

**Affiliations:** ^1^Endocrinology and Diabetology Unit, “M. Bufalini” Hospital, ASL of Romagna, Cesena, Italy; ^2^Internal Medicine Unit, “M. Bufalini” Hospital, ASL of Romagna, Cesena, Italy; ^3^Pathology Unit, “M. Bufalini” Hospital, ASL of Romagna, Cesena, Italy

## Abstract

A 75-year-old man was hospitalized because of severe hypokalaemia due to ACTH dependent Cushing's syndrome. Total body computed tomography (TBCT) and 68 Gallium DOTATATE PET/CT localized a voluminous prostate tumour. A subsequent transurethral prostate biopsy documented a small cell carcinoma positive for ACTH and calcitonin and negative for prostatic specific antigen (PSA) at immunocytochemical study; serum prostatic specific antigen (PSA) was normal. Despite medical treatments, Cushing's syndrome was not controlled and the patient's clinical condition progressively worsened. Surgical resection was excluded; the patient underwent a cycle of chemotherapy followed by febrile neutropenia and fatal intestinal perforation. This case report describes a rare case of Cushing's syndrome and hypercalcitoninaemia due to a small cell carcinoma of the prostate, a rare tumour with very few therapeutic options and negative prognosis.

## 1. Introduction

Ectopic Cushing's syndrome (CS) due to adrenocorticotropic hormone (ACTH) secretion accounts for about 10–15% of all ACTH dependent CS. Ectopic ACTH syndrome is frequently related to small cell carcinomas of the lung or neuroendocrine tumours such as bronchial carcinoids, thymic carcinoids, and pancreatic NETs; more rarely, other neuroendocrine tumours involving gastrointestinal and genitourinary tracts, pheochromocytomas, or medullary thyroid carcinomas may be involved [1, 2]. Plasma calcitonin is a marker of medullary thyroid carcinoma, generally useless in other clinical settings even if sometimes hypercalcitoninaemia can be associated with nonthyroid pathologies or other neuroendocrine tumours [3, 4].

We describe a rare case of Cushing syndrome due to an aggressive small cell prostate carcinoma associated with high levels of plasma calcitonin, an aggressive tumour with very few therapeutic options and a negative prognosis.

## 2. Case Presentation

A 75-year-old man was hospitalized for mental confusion, muscular weakness, and severe hypokalaemia (2.5 mEq/L). The patient's medical history included hypertension and benign prostatic hyperplasia; he took ramipril 5 mg and dutasteride 0.5 mg daily. Few months before he had felt an increasing asthenia, he noted weight gain and the worsening of hypertension control and he experienced some episodes of low urinary tract infections. The laboratory tests evidenced the onset of diabetes (fasting glycaemia 160 mg/dL and HBA1C 50 mmol/L); prostate-specific antigen (PSA) was normal (2.39 *μ*g/L). His wife reported that lastly he was physically exhausted and mentally confused. At physical examination the patient was 165 cm in height and he weighed 70 kgs (BMI 25.7); he showed slight round face and thin arms and legs; his arterial pressure was 160/105 mmHg. Digital evaluation revealed that the prostate was enlarged and firm in consistency. Laboratory tests (Table 1) confirmed severe hypokalaemia (2.3 mmol/L) and documented high levels of midnight salivary cortisol (50.6 *μ*g/L), elevated levels of plasma ACTH (155.4 ng/L), and plasma cortisol (398 *μ*g/L). The overnight 8 mg dexamethasone suppression test did not properly suppress plasma cortisol (198 *μ*g/L); high levels of plasma chromogranin A and calcitonin (272 ng/L) were also documented; PSA was confirmed to be normal (1.7 *μ*g/L) (Table 1).

**Figure 1:**
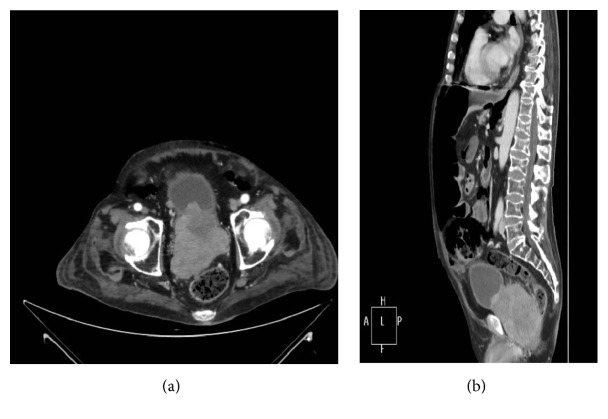
Computed tomography (CT) images, axial (a) and coronal (b), of the pelvis showing a huge mass involving bladder and rectum.

**Figure 2:**
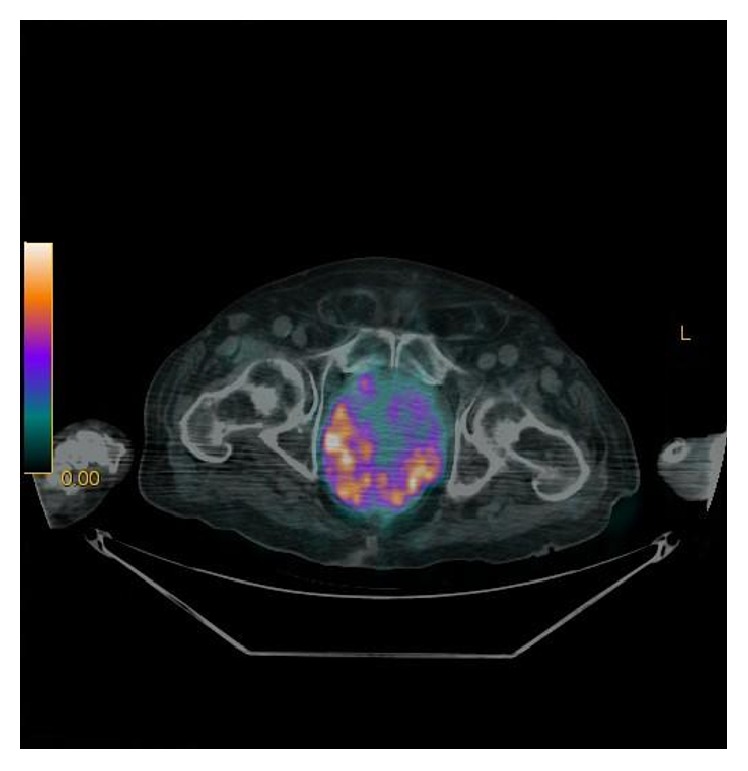
68 Gallium DOTATATE PET/CT image showing a high and diffuse uptake of the prostate.

**Figure 3:**
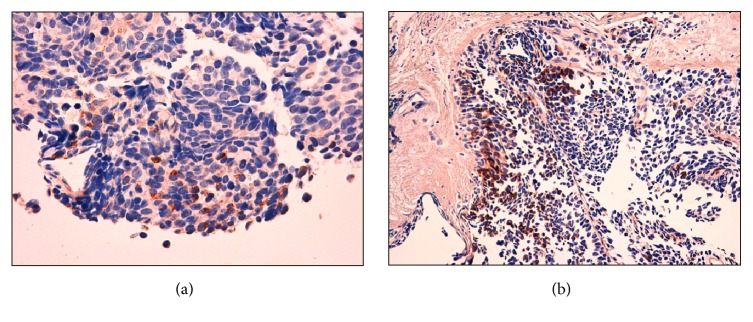
(a) Immunohistochemical stain showing tumour cell positive for ACTH (×40). (b) Immunohistochemical stain showing tumour cell positive for calcitonin (×20).

Magnetic Resonance Imaging (RMI) of the pituitary gland was normal; no nodules were found by thyroid ultrasonography. Total body computed tomography (TBCT) (Figures 1(a)-1(b)) revealed a voluminous prostate gland (maximum diameter of 10 cm) heterogeneously enhancing the contrast medium. The tumour of the prostate had invaded the bladder; the rectum and multiple pathological retroperitoneal lymph nodes as well as bilateral enlarged adrenal glands were evident. A subsequent 68 Gallium DOTATATE PET/CT found a high uptake of the whole prostate (SUV max 12.7) and of the adjacent lymph nodes, confirming the presence of a neuroendocrine tumour in that site and excluding other distant metastases (Figure 2). The patient underwent a transurethral prostate biopsy that showed a small cell prostate cancer focally positive for ACTH and calcitonin and negative for chromogranin A and PSA at immunocytochemistry (Figures 3(a) and 3(b)). Continuous intravenous potassium infusion, potassium sparing drugs (Aldactone), and antihypertensive drugs were administered to the patient. Ketoconazole was also administered with an uptitrating dose of 400 mg/twice daily for 5 weeks and because of the poor control of cortisol secretion, octreotide 0.1 mg every 8 hours subcutaneously was added for further 13 days. Despite a reduction of plasma cortisol (195 mcg/L) after 6 weeks of treatment, no significant clinical benefit was achieved; in fact the patient continued rapidly to worsen becoming more asthenic and confused since he developed a glucocorticoid induced psychosis. The surgeon urologist excluded prostate resection and bilateral surrenectomy because they were considered too dangerous for the patient. The patient started a cycle of chemotherapy with epirubicin and carboplatin, but few weeks after the first cycle, febrile neutropenia, sepsis, and intestinal perforation occurred and the patient died.

## 3. Discussion

Extrapulmonary small cell carcinomas are rare cancers that may involve many tissues. Genital urinary tract (bladder and prostate) and gastroenteric tract are the mostly affected extrapulmonary sites [5]. The prostate is affected in about 10% of all extrapulmonary small cell carcinomas and accounts for 0.2% of all prostate carcinomas [6, 7]. The pathogenesis of the tumour is still poorly understood; it may arise de novo or represent an aggressive terminal phase of a preexisting prostate adenocarcinoma; generally the tumour is not responsive to androgen ablation therapy [6, 8, 9]. In the case described, we suppose a primary form of a neuroendocrine cancer; of note is the fact that the patient was taking dutasteride, an antiandrogen drug known to reduce the risk of prostate cancer [10].

Cushing's syndrome (CS) due to ACTH secreting small cell prostate carcinoma was first described many decades ago [11]; sporadic cases have been reported subsequently, but it remains a very rare and not easily recognized pathology. CS is the consequence of an ectopic secretion of ACTH; hardly and only in this case is it associated with pituitary hyperplasia [12]. To our knowledge, CS of small cell prostate carcinoma has rarely been described in association with high levels of plasma calcitonin [9]; recently CS and hypercalcitoninaemia have been reported in a small cell lung cancer [13]. Small cell carcinoma of the prostate is characterized by poor prognosis because it is quite often metastatic at the diagnosis [7, 14]. Generally PSA is not elevated, in particular in primary type [6], as probably occurring in the patient described. The normality of PSA, which is routinely used in the follow-up of prostatic diseases, may contribute to delay of the diagnosis. It is very aggressive and a rapidly evolving cancer, more resistant to chemotherapy and radiotherapy with respect to the typical adenocarcinoma, showing a high propensity to metastasize. It has been postulated that the poor prognosis is related to the stage of the disease, but the options of cure are limited even in the early stages and, at the moment, there are no defined care plans for this tumour [7]. It has been suggested that TBCT is the most useful first radiological choice in the evaluation of ectopic CS and that a subsequent nuclear medicine modality is requested to confirm or detect a neuroendocrine tumour. Many nuclear medicine imaging modalities like F-FDGPET, octreoscan, or MIBG scintigraphy can be used in order to detect a neuroendocrine tumour, but it has been proven that 68 Gallium-SSTR-PET/CT, when available, can show the highest sensitivity in localizing ectopic ACTH secreting tumours, specially in occult diseases [15]. In particular 68 Gallium DOTATATE, which has a predominant affinity for somatostatin receptors type 2 (SSTR2), can dramatically improve the spatial resolution and lesion detectability compared to octreoscan, MIBG scintigraphy, and F-FDGPET/CT in neuroendocrine tumours and non-GEP-NETs tumours, being mostly accurate in staging patients in whom metastatic cancers spread, particularly to the bone is suspected [16, 17]. 68 Gallium DOTATATE is particularly useful in the early stages when neuroendocrine tumours are generally well differentiated and express significant SSTR2, while F-FDGPET/CT scan may be more appropriate in the late stages when neuroendocrine tumours become poorly differentiated [16].

Radiolabelled somatostatin analogue imaging is a primary indication for neuroendocrine tumours; of note is the fact that prostate cancer expresses all of the five subtypes of somatostatin receptors (SSTR), so gallium labelled somatostatin analogues may be also indicated in the evaluation and staging of this disease [17]. 68 Gallium DOTATATE is considered one of the best modalities to detect neuroendocrine tumours and relative distant metastases useful to guide the choice of the following treatments, directing patients to curative surgery when only a primary site is identified or to systemic therapy when metastases are documented [16]. Beyond the presence of distant metastasis, it has to be considered that, similarly to small cell lung cancer [18], CS is a further marker of poor prognosis because cortisol overproduction induces a rapid worsening of general health. In fact, intense weakness, severe hypokalaemia, metabolic alkalosis, hypertension, hyperglycaemia, and immunodepression are characteristic features at the time of the diagnosis [19] even when the classic signs of CS may be absent, as in the patient described. An early diagnosis and treatment of CS may give a better chance of recovery for the subsequent treatments. To this view, bilateral surrenectomy has been recently reconsidered as a valid treatment in Cushing's disease [20] and it has been proposed as the first therapeutic treatment [19]. Moreover, we must consider that, given the rarity of the disease, no definitive data can be collected and, at the moment, successful therapeutic choices are lacking [5, 19]. Calcitonin is the typical clinical marker of medullary thyroid cancer (MTC) generally useless in other clinical settings even if, rarely, it can be related to other neuroendocrine tumours [3]. Really, it has been proven for decades that calcitonin, as well as other neuroendocrine markers (serotonin, chromogranin A, and enolase neurone specific antigen NSE), is highly expressed in prostate tissue and that the neuroendocrine differentiation of prostate cancer is generally associated with tumours progression and poor prognosis [9, 21]. Recently calcitonin and calcitonin receptor (CTR) have been demonstrated to be highly expressed in advanced grades of prostate carcinoma; calcitonin and CTR are supposed to be involved in a favourable paracrine axis able to induce the progression from a localized to a metastatic cancer [22]. In the case reported here, we can suppose that the presence of high calcitonin serum levels and the positive immunocytochemical stain for calcitonin in the carcinoma can probably be considered a further marker of poor prognosis. Therefore the dosage of plasma calcitonin can be useful not only in the diagnosis of MTC, but even in the evaluation of ectopic ACTH secretion, because high levels of plasma calcitonin can be, although rarely, related to other neuroendocrine tumours.

## 4. Conclusions

ACTH secreting Cushing's syndrome due to small cell prostate cancer is a very rare disease with very few options of cure. Prostate-specific antigen (PSA) is generally not elevated, so finding a normal PSA can be misleading. Gallium labelled somatostatin analogues and in particular 68 Gallium DOTATATE are useful imaging modalities to localize and stage a neuroendocrine tumour of the prostate. In the presence of CS the rapid control of glucocorticoid overproduction must precede the cure of the cancer in order to give a better chance of recovery and a longer survival time for the patient. Bilateral surrenectomy, whenever possible, may be considered a valid therapeutic option.

High levels of plasma calcitonin in the presence of CS may be useful in the differential diagnosis of ACTH dependent CS. Even if it is not possible to drag any conclusion about the role of calcitonin in prostate cancer, on the basis of the case described here, we can suppose that high levels of calcitonin, due to a small cell carcinoma of the prostate, as well as CS, can be related to an unfavourable prognosis.

## Figures and Tables

**Table 1:** Blood test results.

Investigations	Results	Reference range
White blood cell count	12.210	4.00–10.00 10^9^/L
Hemoglobin	15.5	13.5–17.0 g/dL
Creatinine	0.60	0.70–1.2 mg/dL
Potassium	2.5	3.5–5.1 mMol/L
Fasting glucose	160	60–100 mg/dL
Total bilirubin	0.73	<1.2 mg/dL
Alanine aminotransferase	29	<41 U/L
Albumin	31	35–50 g/L
Prostate specific antigen (PSA)	1.7	<4.1 *μ*g/L
Chromogranin A (CGA)	215	<120 *μ*g/L
Plasma ACTH	155.4	7.2–63.3 ng/L
Plasma cortisol	398	62–180 *μ*g/L
Salivary cortisol	50.6	<2.1 *μ*g/L
Testosterone	2.57	4.6–31 nmoli/L
Phosphate	2.6	2.7–4.5 mg/dL
Calcium	8.8	8.6–10.2 mg/dL
Calcitonin	272	<20 ng/L
